# Knowledge-driven teaching-learning-based optimization algorithm for bi-objective flexible job-shop scheduling problem with tool allocation

**DOI:** 10.1371/journal.pone.0342585

**Published:** 2026-02-17

**Authors:** Kuineng Chen, Xiaofang Yuan, Weihua Tan

**Affiliations:** 1 Hunan Engineering Research Center of Special Robot Control Technology and Equipment in Complex Environment, Xiangtan, China; 2 College of Electrical and Information Engineering, Hunan University, Changsha, China; 3 Sanya Institute of Hunan University of Science and Technology, Sanya, China; 4 School of Information and Electrical Engineering, Hunan University of Science and Technology, Xiangtan, China; Memorial Sloan Kettering Cancer Center, UNITED STATES OF AMERICA

## Abstract

To perform “global” optimization of the machining process in discrete manufacturing, a bi-objective flexible job-shop scheduling problem with tool allocation is proposed. Unlike traditional scheduling problems that treat resources independently, this paper addresses the strong coupling between machine routing, operation sequencing, and finite tool capacity. A mixed-integer programming model is constructed with the objectives of minimizing the tool wear cost and weighted sum of tardiness. Sophisticated constraints that fit actual manufacturing scenarios are considered, specifically the combination of tool magazine capacity, variant job releasing times, and machine/tool compatibility for operations. To address the computational challenge and the discrete nature of the solution space, a knowledge-driven teaching-learning-based optimization algorithm is designed. Specific strategies, including a topology-preserving discrete crossover and a critical-path-based neighborhood search, are developed to prevent premature convergence caused by complex constraints. Simulation experimental results show that the proposed algorithm significantly outperforms the traditional meta-heuristic algorithms in the aspects of quality, spread, and comprehensive metric, and the proposed multi-objective collaborative optimization method obtains better processing decisions than the traditional sequential scheduling methods.

## 1 Introduction

Effective production scheduling plays a fundamental role in the modern discrete manufacturing system, due to its great potential to promote efficiency and productivity. With the increasing demand for customized manufacturing that is generally characterized by the short life cycle of product, small lot size, and rapidly changing product mix, the flexible job shop has drawn great research attention in the last decades [1]. In flexible job-shop environment, the machine accommodates multiple kinds of operations [2], thanks to diverse tools allocated in the tool magazine. To rapidly and cost-effectively reconfigure machine functions, the modular design of tools are commonly adopted, allowing for flexible adaptation across multiple machines through standardized software and hardware interfaces. Thus, there arise an operational challenge to providing appropriate tool allocation. Obviously, the decisions regarding tool allocation have influence on the scheduling. The machine functions is directly related to tool allocation, thereby determining various production indicators such as processing time, and costs. To provide global decisions, it is necessary to integrate the optimization of tool allocation in the flexible job shop scheduling problem.

Tools are crucial components in FMS environments due to their high cost. According to [3], tooling accounts for approximately 25-30% of production costs. The optimization problem becomes more intricate with the additional decisions related to tool allocation. On one hand, numerous constraints are closely related to tools, such as tool capabilities [4] and the limitations on the number of tools a machine can hold [5,6]. Once the tools are set, the configuration of each magazine should not be changed, so each machine can only employ the tool copies already allotted in its magazine. The tool allocation is coupled with the production scheduling including machine assignment, operation sequence [7]. One the other hand, the attributions of tool have impacts on the performance of shop. As mentioned by [8], the tool property will change with time and tool needs reconditioning or replacement before they are wear out. Therefore, tool life-time needs to be considered in the scheduling problem. As analysis before, the tool allocation is a key factor for scheduling optimization. However, no research effort has considered this issue in flexible job shop environment yet, which will lead to sub-optimal solution for the scheduling decision.

Consequently, tool allocation is deeply coupled with production scheduling [7]. Unlike parallel machine environments where jobs are assigned to a single resource, the routing flexibility in job shop means that machine assignment decisions vary dynamically based on the available tool slots in different magazines. On the other hand, the attributions of tool have impacts on the performance of shop. As mentioned by [8], the tool property will change with time and tool needs reconditioning or replacement before they are wear out. Therefore, tool life-time needs to be considered in the scheduling problem. As analysis before, the tool allocation is a key factor for scheduling optimization. However, the majority of existing literature focuses on tool allocation in simpler environments such as parallel machines or assembly lines. Research that simultaneously considers the flexible routing, strict tool magazine capacity limits, and dynamic job releasing times in a unified framework is scarce. Neglecting these coupled constraints in flexible job shop leads to sub-optimal or even infeasible solutions for actual manufacturing decisions.

Addressing the shortcomings of the aforementioned research, this paper focuses on the bi-objective flexible job-shop scheduling problem with tool allocation (BFJSP-TA). The contributions of this paper are threefold:

1) An mixed-integer programming model of BFJSP-TA is constructed with the objective of optimizing both tool wear cost and weighted sum of tardiness. The model explicitly formulates the hard constraints of tool magazine capacity, variant job releasing times, and machine/tool compatibility, bridging the gap between theoretical flexible job shop models and physical tooling restrictions.
2) A knowledge-driven teaching-learning-based optimization algorithm is developed. Improvement strategies, including chaotic initialization, and discrete adaptation mechanisms such as crossover-based teacher-learner operators and critical-path-based neighborhood search, are proposed to overcome the lack of structural information in continuous operators and guide the search towards the Pareto front.
3) The necessity of considering tool is validated, and the scenarios with constrained and enriched tools are enclosed in discussion.

The remainder of this paper is organized as follows. Sect 2 provides a critical review of the current literature and highlights the differences between this work and existing studies. Sect 3 describes the problem description and mathematical model of the proposed BFJSP-TA. The teaching-learning-based optimization algorithm enhanced via problem-specific knowledge is detailed in Sect 4. In Sect 5, the comprehensive experiments are presented and an in-depth discussion on the results is provided. Sect 6 presents concluding remarks and enlightens the future research opportunities.

## 2 Literature review

As highlighted in a recent comprehensive review by Dauzère-Pérès et al. [1], the research on the flexible job shop scheduling problem (FJSP) is evolving from static environments to those incorporating complex constraints and multi-criteria optimization, reflecting real-world manufacturing requirements. Effective allocation of tools has become a pivotal issue in the field of discrete manufacturing. Several studies have incorporated the effects of tool degradation. Tian et al. [9] developed a model to understand the correlation between the degradation of cutting tools and the energy expenditure in manufacturing, addressing the production scheduling challenges within a flexible job shop environment with consideration of tool wear. An et al. [10] explored the optimization of maintenance and production scheduling in tandem, focusing on the impact of tool degradation to minimize overall workshop energy consumption. More recently, Wang et al. [11] extended this line of research to distributed manufacturing, proposing an NSGA-III based approach for rescheduling that explicitly considers cutting tool maintenance. However, their work focuses on the distributed flow shop rather than the tightly coupled tool capacity constraints within a single flexible job shop.

In numerous practical manufacturing settings, the management of tools is intricately linked to the decision-making processes of production. Recently, there has been a surge in interest in the multi-objective optimization of production processes that involve tools. Atta et al. [12] refined the assignment of tools to CNC machine tool slots, employing an enhanced harmony search algorithm. Reddy et al. [13] and colleagues delved into the optimization of the synergistic operation of machining equipment, tools, and automated guided vehicles, while Zhang et al. [3] concentrated on production optimization scheduling that accounts for tool changeover time and machining rates.

Regarding the production environment, the majority of existing studies have concentrated on the production demands of assembly lines, examining the scenarios of parallel machines and non-identical parallel machines. However, research that targets the more adaptable job shop environments with strictly limited tool magazine capacities is comparatively scarce.

### 2.1 Advances in teaching-learning-based optimization algorithm

The Teaching-Learning-based Optimization (TLO) algorithm was proposed by Rao in 2011 [14], which is a metaheuristic algorithm that mimics the teaching and learning behaviors in a classroom setting. Due to its simple structure and excellent performance, the TLO algorithm has been widely applied to practical problems since its inception. In the context of optimization issues in turning processes, Lin et al. proposed a multi-objective TLO algorithm that employs an improved random key encoding method and uses solutions from the non-dominated set as the “teacher solution” in the teaching phase. Li et al. [15] defined the optimal solution based on the dominance relationship and further combined the Sigma method to rank solutions in the non-dominated set, thus distinguishing the quality of non-dominated solutions. Shao et al. [16] developed a discrete TLO algorithm that directly encodes solutions in the discrete space and introduced a probabilistic model to control the learning intensity during the learning phase. Currently, the application of TLO algorithms to discrete problems is mainly achieved by defining a mapping relationship between the discrete and continuous spaces for solution encoding and subsequent evolutionary operations.

Recent advancements have further demonstrated the potential of TLO and similar evolutionary mechanisms in complex scheduling. Tan et al. [17] proposed an enhanced multi-objective TLBO for integrated production and distribution scheduling, verifying that incorporating heuristic rules and specific neighborhood searches can significantly boost performance in bi-objective problems. Similarly, in the broader context of evolutionary computation, Ding et al. [18] integrated a fluid master-apprentice evolutionary algorithm with deep reinforcement learning to solve the FJSP with multiplicity, highlighting the growing trend of utilizing learning mechanisms to guide the evolutionary process. Furthermore, Luo et al. [19] developed an affinity propagation hierarchical memetic algorithm for multimodal multi-objective FJSP, employing problem-specific neighborhood structures to enhance convergence. These studies collectively suggest that generic operators are often insufficient for complex constraints.

However, due to the difficulty in reflecting the structural information of the discrete space in the continuous space, the standard mapping-based approach often disrupts the precedence constraints of scheduling. At the same time, utilizing problem knowledge to guide the search direction of the TLO algorithm, thereby reducing the randomness of the evolutionary direction, is of great significance for solving the multi-objective collaborative optimization problem of the tool and processing equipment in the discrete manufacturing environment.

### 2.2 Brief summary

Despite the advancements, there are notable deficiencies in the current research. First, The lack of research on job shop environments is a significant oversight compared to parallel machine settings, given their prevalence in modern manufacturing. The existing models have made significant strides in incorporating tool degradation effects and optimizing maintenance alongside production scheduling. However, these models often rely on complex assumptions that may not fully capture the nuances of real-world manufacturing scenarios where tool magazine capacity is strictly finite and strongly coupled with machine routing flexibility. The integration of tool wear, energy consumption, and production efficiency into a cohesive model remains a challenge, with the potential for oversimplification or over complication hindering practical applicability. The literature pertinent to scheduling problem with tool allocation published in recent years are summarized in Table 1.

**Table 1:** Summary of literature pertinent to scheduling problem with tool allocation published in recent years.

Paper	Production environment^a^			Objective^b^				Constraints^c^				
	PM	FS	FJS	Tardiness	TWC	Makespan	other	TMC	RT	TL	TN	Capability
[8]			√			√		√		√	√	
[20]	√					√				√		
[21]	√			√		√	√			√		
[22]	√					√						
[23]		√					√					
[3]	√			√		√	√			√		
[10]			√	√		√	√		√			
[6]	√			√			√	√	√			
[9]			√			√	√					
		√			√	√	√			√		
This work			√	√	√			√	√	√	√	√

^a^ PM: parallel machines; FS: flow shop; FJS: flexible job shop.

^b^ TWC: tool wear cost

^c^ TMC: tool magazine capacity; RT: release time; TL: tool life-time; TN: tool number.

Additionally, the reliance on mapping techniques in TLO algorithms for discrete problems limits the effectiveness of these solutions. There is a clear need for more research that can bridge the gap between theoretical models and practical applications, particularly in developing algorithms that can effectively handle the discrete nature of tool allocation problems without losing critical structural information. Furthermore, the incorporation of problem-specific knowledge to guide the search process in TLO algorithms could significantly enhance the solution quality and convergence speed, a direction that current research has yet to fully explore.

## 3 Problem description and formulation

### 3.1 Definition of BFJSP-TA

**Fig 1:**
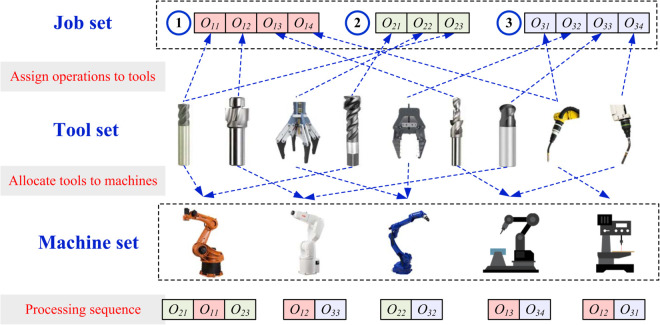
Scenario of the proposed BFJSP-TA.

The scenario of the proposed BFJSP-TA is illustrated in Fig 1, which can be defined as follows. There is a set of machines ***M*** and a set of tools ***K***. Each tool k∈K is treated as an individual tool copy and is assumed to be compatible with all machines. During the planning horizon, each tool copy is assigned to at most one machine, and the tool magazine configuration is assumed to remain unchanged. There is a set of jobs ***J*** need to be processed. The release time and due time of job *i* are *A*_*i*_ and *D*_*i*_, respectively. The job i∈J contains a set of operations $O_{i}$ with precedence constraint. Notably, three important characters of real-world manufacturing scenarios are considered as follows:

- Machine *m* has a limited tool slot capacity *Q*_*m*_.
- The punishment on tardiness of jobs are different.
- Operation *O*_*ij*_ has compatible machine set *M*_*ij*_ and tool set *K*_*ij*_.

Notations of the proposed BFJSP-TA are listed in Table 2. To simplify the problem at hand, the following predefined hypothesis should be satisfied: *a*) transportation and setup time is negligible, *b*) preemption of jobs is not allowed, *c*). all machines and tools are available at the beginning, *d*) breakdown is not considered throughout the planning horizon. These assumptions are commonly adopted in the flexible job-shop literature [24,25] to focus on the coupled decisions of tool allocation and production scheduling, while avoiding additional uncertainties and auxiliary times that would require extra parameters and decision variables. Therefore, the BFJSP-TA contains three sub-problems: tool assigning to machine, operation assigning to machine and tool, operation sequencing. The goals of BFJSP-TA are minimizing the tool wear cost and weighted sum of tardiness, simultaneously.

**Table 2:** Notations of BFJSP-TA.

Notation	Description
*Indices:*	
$i,i^{\prime }$	Job index
$j,j^{\prime }$	Operation index
*m*	Machine index
*k*	Tool index
*p*	Index of the position on machine
*Sets:*	
***J***	Set of jobs
***M***	Set of machines
***K***	Set of tools
$O_{i}$	Set of operations of job *i*, i∈J
*Parameters:*	
$\omega_{i}$	Tardiness weight of job *i*, i∈J
$A_{i}/D_{i}$	Release time/due time of job *i*, i∈J, $A_{i}\geq 0$
*O*_*ij*_	The *j*^*th*^ operation of job *i*, $i\in \text{J},j\in O_{i}$
*M*_*ij*_	Compatible machines for *O*_*ij*_, $i\in \text{J},j\in O_{i},M_{ij}\subset \text{M}$
*K*_*ij*_	Compatible tools for *O*_*ij*_, $i\in \text{J},j\in O_{i},K_{ij}\subset \text{K}$
*T*_*ij*,*k*,*m*_	Processing time for *O*_*ij*_ on machine *m* with tool *k*, $i\in \text{J},j\in O_{i},k\in K,m\in M$
$\xi_{t}$	Cost coefficient of tool *k*, k∈K
$\nu_{ij,k}$	Wear coefficient of tool *k* processing *O*_*ij*_, $i\in \text{J},j\in O_{i},k\in K$
*Q*_*m*_	Tool slot capacity of machine *m*, m∈M
$\delta_{m}$	Number of operations assigned to machine *m*, m∈M
*L*	A very big positive real number
*Decision variables:*	
*c*_*ij*_	Completion time of the *O*_*ij*_, $i\in \text{J},j\in O_{i}$
*X*_*ij*,*k*,*m*_	If operation *O*_*ij*_ is processed by tool *k* on machine *m* , *X*_*ij*,*k*,*m*_=1;
	otherwise, *X*_*ij*,*k*,*m*_=0, $i\in \text{J},j\in O_{i},k\in K,m\in M$
*Y*_*ij*,*m*,*p*_	If operation *O*_*ij*_ is processed at the *p*^*th*^ position on machine *m*, *Y*_*ij*,*m*,*p*_=1;
	otherwise, *Y*_*ij*,*m*,*p*_=0, $i\in \text{J},j\in O_{i},m\in M,p\in \delta_{m}$
*Z*_*k*,*m*_	If tool *k* is assigned to machine *m*, *Z*_*k*,*m*_=1; otherwise, *Z*_*k*,*m*_=0, k∈K,m∈M
*C*_*i*_	Completion time of job *i*, i∈J
*Objectives:*	
*TWC*	Tool wear cost
*WST*	Weighted sum of tardiness

### 3.2 Mathematical model of BFJSP-TA

*a. Formulation of tool wear cost (TWC)*

Using a tool for machining will inevitably result in tool wear [8], and the cost associated with this wear is directly proportional to the duration of the tool’s usage. Therefore, the cost of tool wear can be calculated using Eq. 1.

$$TWC=\sum_{k\in K}\xi_{t}\left\{\sum_{i\in \text{J}}\sum_{j\in {\text{O}}_{i}}\left[\nu_{ij,k}\sum_{m\in M}(T_{ij,k,m}X_{ij,k,m})\right]\right\}$$ (1)

*b. Formulation of weighted sum of tardiness (WST)*

In this study, tardiness is defined as the positive deviation of a job’s completion time from its due time, i.e., the amount of time a job finishes after its due date. To reflect that different jobs may have different priorities in a practical machine shop, the weighted sum of tardiness is adopted, where each job *i* is assigned a tardiness weight $\omega_{i}$ and higher-priority jobs incur larger penalties when delayed. Accordingly, the weighted sum of tardiness aggregates the weighted tardiness of all jobs in the schedule, capturing the overall impact of delays under their respective priorities.

The calculation of the weighted sum of tardiness involves determining the tardiness for each job by subtracting the due date from the completion time, assigning a weight to each job based on its priority or the cost of delay, and then multiplying the tardiness by its weight to obtain the job’s weighted tardiness. The completion time is obtained by Eq. 2, and therefore, the weighted sum of tardiness is calculated by Eq. 3.

$$C_{i}=\max_{j\in {\text{O}}_{i}}\left\{c_{ij}\right\},\;i\in \text{J}$$ (2)

$$WST=\sum_{i\in \text{J}}\omega_{i}\max\left\{C_{i}-D_{i},0\right\}$$ (3)

*c. Mixed-integer programming (MIP) model of BFJSP-TA*

The MIP model of BFJSP-TA can be formulated as follows.

$$minimize\;\left\{ \begin{array}{c} f_{1}=TWC\\ f_{2}=WST \end{array}\right.$$ (4)

Subject to:

$$\sum_{k\in K}\sum_{m\in M}X_{ij,k,m}=1,\;i\in \text{J},j\in {\text{O}}_{i}$$ (5)

$$\sum_{m\in M}Z_{k,m}\leq 1,\;k\in K$$ (6)

$$X_{ij,k,m}=0,\;i\in \text{J},j\in {\text{O}}_{i},k\notin K_{ij},m\in M$$ (7)

$$X_{ij,k,m}=0,\;i\in \text{J},j\in {\text{O}}_{i},k\in K,m\notin M_{ij}$$ (8)

$$\sum_{m\in M}\sum_{p\in \delta_{m}}Y_{ij,m,p}=1,\;i\in \text{J},j\in {\text{O}}_{i}$$ (9)

$$\sum_{i\in \text{J}}\sum_{j\in {\text{O}}_{i}}Y_{ij,m,p}\leq 1,\;m\in M,p\in \delta_{m}$$ (10)

$$\sum_{i\in \text{J}}\sum_{j\in {\text{O}}_{i}}Y_{ij,m,p}\geq \sum_{i^{\prime }\in \text{J}}\sum_{j^{\prime }\in {\text{O}}_{i^{\prime }}}Y_{i^{\prime }j^{\prime },m,p+1},\;m\in M,p,p+1\in \delta_{m}$$ (11)

$$\sum_{k\in \text{K}}Z_{k,m}\leq Q_{m},\;m\in M$$ (12)

$$\sum_{k\in K}X_{ij,k,m}=\sum_{p\in \delta_{m}}Y_{ij,m,p},\;i\in \text{J},j\in {\text{O}}_{i},m\in M$$ (13)

$$Z_{k,m}\geq X_{ij,k,m},\;i\in \text{J},j\in {\text{O}}_{i},k\in K,m\in M$$ (14)

$$c_{ij}\geq \sum_{k\in K}\sum_{m\in M}T_{ij,k,m}X_{ij,k,m},\;i\in \text{J},j\in {\text{O}}_{i}$$ (15)

$$c_{ij}-\sum_{k\in K}\left(T_{ij,k,m}X_{ij,k,m}\right)\geq c_{i\left(j-1\right)},\;i\in \text{J},(j-1),j\in {\text{O}}_{i},m\in M$$ (16)

$$c_{ij}-\sum_{k\in K}\left(T_{ij,k,m}X_{ij,k,m}\right)\geq A_{i},\;i\in \text{J},j\in {\text{O}}_{i},m\in M$$ (17)

$$c_{i^{\prime }j^{\prime }}-\sum_{k\in K}\left(T_{i^{\prime }j^{\prime },k,m}X_{i^{\prime }j^{\prime },k,m}\right)+L\left[1-\sum_{k\in K}\left(Y_{ij,m,p}Y_{i^{\prime }j^{\prime },m,p+1}\right)\right]\geq c_{ij},\;\;i,i^{\prime }\in \text{J},j\in {\text{O}}_{i},j^{\prime }\in {\text{O}}_{i^{\prime }},m\in M,p,p+1\in \delta (m)$$ (18)

Eq. 4 is the objective function. Constraint 5 denotes that each operation is processed only once. Constraint 6 indicates that each tool copy is assigned to at most one machine during the planning horizon. Constraints 7 and 8 guarantee that the operation can only be processed by the compatible machine and tool, respectively. Constraints 9 - 11 denote that the operations are sequenced on the machine one by one. Constraint 12 guarantees the tool slot capacity of each machine. Constraints 13 and 14 ensure no conflicts between the decision variables. Constraint 15 links the completion time of each operation to its selected processing time under the chosen machine–tool pair. Constraint 16 enforces the precedence relationship of the operations belonging to the same job. Constraint 17 indicates that the job can not be processed before the release time. Constraints 18 enforces machine capacity by ensuring that two operations assigned to two consecutive positions on the same machine are processed without overlap.

## 4 Knowledge-driven teaching-learning-based optimization algorithm

When employ the traditional teaching-learning-based optimization (TLBO) algorithm to solve the proposed BFJSP-TA, several intrinsic structural mismatches and convergence challenges arise. First, the continuous nature of standard TLBO operators disrupts the discrete precedence constraints of scheduling, leading to infeasible offspring. Furthermore, standard evolutionary operators lack the domain-specific guidance required to navigate the strongly coupled constraints of tool capacity and routing flexibility, often resulting in stagnation at local optima. To tackle these issues, a knowledge-driven teaching-learning-based optimization (KTLBO) algorithm is designed. The KTLBO aims to bridge the gap between evolutionary search and discrete scheduling logic by incorporating problem-specific knowledge, specifically critical path properties and tool cost indicators, into the teaching-learning process. The overall procedure of KTLBO is depicted in Algorithm 1, and the specific improvement strategies are detailed in the following sections.

**Algorithm 1 Overall procedure of KTLBO.**

**Require:** npop, termination time

1: **Input:** Population size npop, algorithm termination time

2: **Output:** First front solution set F1

3:   Initialize the population P ▷ Refer to Sect 4.2

4: **while** termination time not reached **do**

5:    C ← DiscreteTeachAndLearn(P) ▷ Discrete teaching and learning operator, refer to Sect 4.3

6:    P ← Merge_DiscardRep(P, C) ▷ Merge population and discard duplicates

7:    P ← NeighborSearch(P) ▷ Neighborhood search based on the movement of critical operations, refer to Sect 4.4

8:    Ps ← NonDominateSort(P) ▷ Non-dominated sorting of P to determine the non-dominated front, stored in Ps

9:    Pr ← CrowdDistanceRank(Ps) ▷ Ranking solutions in the same non-dominated front by crowding distance, stored in Pr

10:    P ← Pr(1:npop)

11: **end while**

12:   P ← NeighborSearch(P) ▷ Non-dominated sorting of P to determine the non-dominated front, stored in Ps

13:   F1 ← Ps{1} ▷ Store the first non-dominated front solutions in F1

14: **return** F1

### 4.1 Solution encoding and decoding

**Fig 2:**
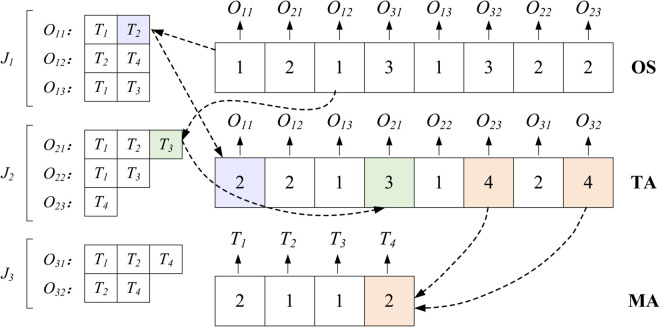
Illustration of the proposed coding method.

The encoding method must ensure the feasibility of solutions and facilitate subsequent evolutionary operations of the algorithm. For the BFJSP-TA problem, this paper designs a three-row vector encoding method. The three-row vector includes: the Operation Sequence (OS) vector, the Tool Allocation (TA) vector, and the Machine Allocation (MA) vector. As illustrated in Fig 2, the encoding example and specific descriptions of these vectors are as follows:

- **OS vector**: This vector represents the sequence of operations from left to right, with its length equal to the number of operations to be processed. Each element is an operation, denoted by the workpiece index, and elements with the same operation index are arranged from left to right for all operations of the same workpiece.
- **TA vector**: The elements of this vector correspond one-to-one with the operations to be processed, and this mapping relationship remains unchanged throughout the entire evolutionary process of the algorithm. The element value represents the tool allocated to the corresponding operation, thus the length of the TA vector is equal to the number of operations to be processed.
- **MA vector**: The elements of this vector correspond one-to-one with the available tools, and this mapping relationship remains unchanged throughout the entire evolutionary process of the algorithm. The element value represents the machine allocated to the corresponding tool, thus the length of the MA vector is equal to the number of available tools.

This paper adopts an “active decoding” method [17] for decoding, which can be described as follows: Arrange the operations according to the OS vector from left to right; for operation *O*_*ij*_, allocate *O*_*ij*_ to tool *K*_*k*_ according to the corresponding element value in the TA vector, thus determining its processing time, and determine the machine *M*_*m*_ for *O*_*ij*_ according to the corresponding element value in the MA vector (i.e., the machine allocated to tool), placing the operation at the earliest possible start time without affecting the start time of other operations and without violating constraints.

### 4.2 Chaotic initialization

**Fig 3:**
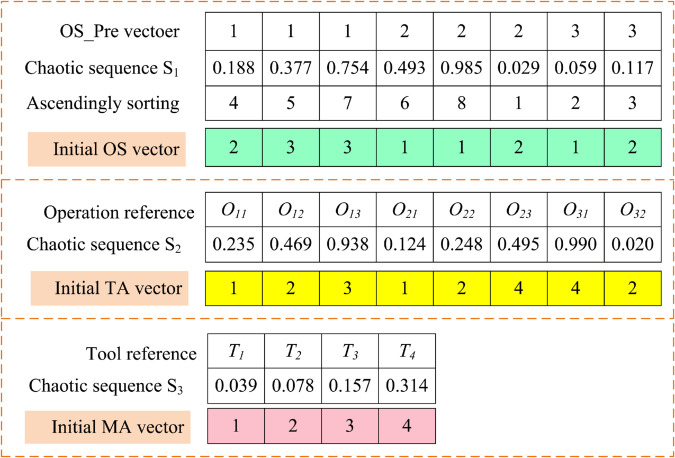
Illustration of the proposed chaotic initialization method.

Generally, enhancing the diversity of solutions in the initial population helps to reduce the probability of the algorithm falling into a local optimum during the evolutionary process [26]. Chaotic mapping has a series of characteristics such as initial value sensitivity and pseudo-randomness, and has been widely used in existing literature to enhance the diversity of the algorithm’s population [27,28]. Therefore, this paper combines the Tent chaotic mapping [29] to generate initial solutions. The specific steps are as follows:

Step 1: Randomly generate a number *g* within the range of 0 to 1.
Step 2: Using *g* as the initial value of the chaotic sequence, generate a chaotic sequence *S*_1_ with the same length as the OS vector using the Tent chaotic mapping, and rearrange the preset OS_Pre vector order according to the size of *S*_1_; as shown in Fig 3, the smallest number in *S*_1_ corresponds to 2 in OS_Pre, so the first element of the OS vector is 2, and similarly, the initial OS vector is obtained.
Step 3: Using the last number of *S*_1_ as the initial value of the chaotic sequence, generate a chaotic sequence *S*_2_ with the same length as the TA using the Tent chaotic mapping, and use a linear mapping method to associate the chaotic sequence with the optional tool number corresponding to the element, thus obtaining the initial TA vector; if using the tool configuration available for the operation in Fig 2, the initial TA vector can be obtained as shown in Fig 3, for example: the third number of *S*_2_ is 0.938, corresponding to the second optional tool of *O*_12_, then it denotes *k*_3_.
Step 4: Using the last number of *S*_2_ as the initial value of the chaotic sequence, generate a chaotic sequence *S*_3_ with the same length as the TA using the Tent chaotic mapping, and the subsequent process is very similar to the initialization of OS, which is not repeated here.

### 4.3 Crossover-based teacher and learner operators

The traditional Teaching-Learning Optimization (TLO) algorithm is designed for single objective and continuous optimization problems, and its specific process can be found in the literature [14]. To effectively apply the evolutionary mechanism of the TLO algorithm to the multi-objective discrete optimization problems presented in this paper, two aspects require to be emphasized:

- How to determine the teacher solution? In the traditional TLO, the solution with the best fitness in the current population is chosen as the teacher solution. However, in multi-objective optimization problems, there are usually multiple non-dominated front solutions, making the original approach inapplicable.
- How to exchange information between solutions? In this paper, the encoding of solutions consists of discrete element combinations, which makes it difficult to use the numerical operation methods in traditional TLO for information exchange.

**Fig 4:**
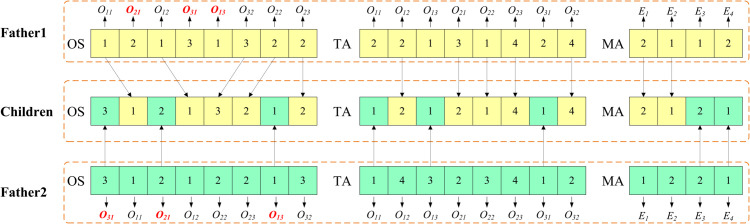
Scheme of MPX.

To address these issues, this paper uses the crowding degree in the objective space [30] to rank solutions that are on the same non-dominated front and introduces a teacher ratio parameter rT to determine the proportion of teacher solutions in the population. A multi-point crossover (MPX) operation is used to achieve information exchange between two solutions, and a crossover intensity parameter ζ is introduced. The schematic diagram of the MPX operation is shown in Fig 4, where the OS vector uses a position-based multi-point crossover method, and the TA and MA vectors use a direct multi-point crossover method, with the number of crossover points being the crossover intensity ζ multiplied by the vector length. The overall process of the discrete teaching and learning operator is described in the pseudo-code of Algorithm 2.

**Algorithm 2 Procedure of the crossover-based teaching and learning operator.**

**Require:** Population *P*, number of population members *npop*, teacher ratio *r*_*T*_, crossover intensity ζ

1: *Ps* ←NonDominateSort(P) ▷ Non-dominated sorting of *P* to determine the non-dominated front, stored in *Ps*

2: Pr←CrowdDistanceRank(Ps) ▷ Ranking solutions in the same non-dominated front of *Ps* by crowding distance, stored in *Pr*

3: $\text{nT}\leftarrow \lceil \text{npop}\times {\text{r}}_{\text{T}}\rceil$ ▷ Calculating the number of teacher solutions

4: Te←Pr(1:nT),St←Pr(nT+1:end) ▷ The first *nT* in *Pr* form teacher solution set, the rest form student solution set

5: **for** n=1→size(St) **do**

6:   te←SelRand(Te,1) ▷ Randomly select a teacher solution *te* from the set of teacher solutions *Te*

7:   Cte(n)←MPX(St(n),te,ζ) ▷ A student learns from a teacher via the MPX operator, and its offspring is stored in *Cte*

8: **end for**

9: **for** n=1→size(St) **do**

10:   st←SelRand(St,1) ▷ Randomly select a student solution *st* from the set of student solutions *St* such that st≠St(n)

11:   Cst(n)←MPX(St(n),st,ζ) ▷ A student learns from a teacher via the MPX operator, and its offspring is stored in *Cst*

12: **end for**

13: C←Merge_DiscardRep(Cte,Cst) ▷ Merge offspring solutions from the teaching and learning phases, and discard duplicates

14: **return** *C*

### 4.4 Neighborhood search

Critical operations are one of the key pieces of knowledge in the collaborative optimization of production processes, and traditional movement of critical operations can quickly reduce completion time [29]. In response to the two objectives of BFJSP-TA, KTLBO has improved upon the traditional movement of critical operations, designing an efficient local search strategy.

**Fig 5:**
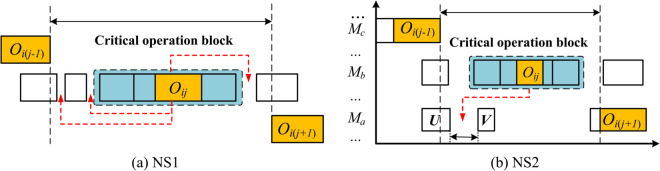
Illustration of the proposed neighborhood structures.

The basic idea behind the traditional movement of critical operations is to remove the critical operation from its current critical operation block, and the schematic diagram of its movement is shown in Fig 5. The specific process has been described in detail in the literature [29] and will not be reiterated here. This paper has made improvements in two aspects:

Population Grouping: Local search can easily reduce the diversity of the population, leading to the algorithm falling into a local optimum. To balance the intensity of search and the diversity of the population, the population is evenly divided into three groups, namely: one that only executes the Neighborhood Structure 1 (NS1), one that only executes neighborhood structure 2 (NS2), and one that executes NS1 and NS2 with equal probability;

Critical Operation selection: To balance the production cost and completion time objectives during the evolution, the selection of the critical operation to be moved will be biased towards operations that can quickly reduce their production cost. The specific approach is to identify those operations whose current processing cost is greater than their average production cost (the average of production costs corresponding to all possible tool allocation schemes), and then randomly select one of them for movement.

## 5 Computational experiments and discussion

In this section, the performance validation of the proposed improvement strategies and the overall performance of the KTLBO algorithm are presented, and necessity of the proposed collaborative optimization method are analyzed.

The discrete manufacturing domain-specific benchmarks DP13-18, MK06, MK08-10, MK12-15 are modified to generate instances suitable for the BFJSP-TA (renamed as #DP13-18, #MK06, #MK08-10, #MK12-15). These widely recognized benchmarks are selected for their representativeness in capturing the topological complexity and diversity of flexible job shops. Specifically, they cover varying problem scales, ranging from small-scale problems to large-scale complex scheduling tasks, allowing for an assessment of the algorithm’s scalability. More importantly, strict physical constraints encountered in actual workshops, such as limited tool magazine slots and distinct tool wear rates, are explicitly integrated into these instances to simulate realistic manufacturing resource contentions.

Generational Distance (GD), Diversity (Δ), and Inverted Generational Distance (IGD) are chosen as the evaluation metrics for the quality of the front solutions, with their definitions provided in reference [31]. Each experiment is independently run 20 times to eliminate randomness. To ensure fairness in comparison, the computation time is set to be the same for all algorithms. All algorithms involved in this paper are programmed on the Matlab R2018b platform, and the simulation experiments are conducted on a computer with an Intel Core i7 (3.0GHz) processor, Windows 10 operating system, and 8GB of memory. To align with the reproducibility requirement, the specific parameter settings for the comparison algorithms (NSGA-II, MOGWO, and SPEA2) are adopted from their original literature or standard settings, as detailed in Table 3. The following sections detail the simulation experiment process and analyze the results.

**Table 3:** Parameter settings of the comparative algorithms.

Algorithm	Parameter	Value
NSGA-II	Crossover probability (*p*_*c*_)	0.9
	Mutation probability (*p*_*m*_)	1/*n* (*n* is the number of variables)
	Distribution index ($\eta_{c},\eta_{m}$)	20, 20
MOGWO	Grid inflation parameter (*α*)	0.1
	Number of grids (*n*_*grid*_)	10
SPEA2	Archive size	50

### 5.1 Parameter tuning

The KTLBO algorithm includes three important parameters: population size (npop), teacher solution ratio (rT), and crossover intensity (ζ). We used orthogonal experiments to optimize these parameters, selecting 4 values within a reasonable range for each parameter, which were determined based on preliminary tests. The experiments are arranged using the orthogonal table $L_{16}(4^{3})$. To eliminate the impact of randomness inherent in meta-heuristics, each experimental configuration in the orthogonal array was executed 20 times independently to obtain the average IGD value.

The experiments are conducted on the #DP18 test case, using IGD as the comprehensive evaluation metric. The design of the orthogonal experiments and the results are shown in Table 4.

**Table 4:** Orthogonal experiment results for parameter tuning and range analysis.

ID	npop	rT	ζ	IGD
1	10	0.1	0.01	0.0103
2	10	0.2	0.05	0.0047
3	10	0.3	0.1	0.0034
4	10	0.5	0.2	0.0057
5	30	0.1	0.05	0.0035
6	30	0.2	0.01	0.0059
7	30	0.3	0.2	0.0036
8	30	0.5	0.1	0.0053
9	80	0.1	0.1	0.0099
10	80	0.2	0.2	0.0100
11	80	0.3	0.01	0.0077
12	80	0.5	0.05	0.0040
13	200	0.1	0.2	0.0080
14	200	0.2	0.1	0.0071
15	200	0.3	0.05	0.0059
16	200	0.5	0.01	0.0066
*Range Analysis:*				
*K*_1_ (Avg.)	0.0060	0.0079	0.0076	
*K*_2_ (Avg.)	**0.0046**	0.0069	**0.0045**	
*K*_3_ (Avg.)	0.0079	**0.0052**	0.0064	
*K*_4_ (Avg.)	0.0069	0.0054	0.0068	
Range (*R*)	0.0033	0.0028	0.0031	

To determine the detailed impact of each parameter, the Taguchi method is employed. As presented in the bottom of Table 4, *K*_*i*_ denotes the average IGD value for a specific factor at level *i*, and the Range ($R=\max(K_{i})-\min(K_{i})$) indicates the sensitivity of the parameter. A smaller *K*_*i*_ signifies better performance, while a larger *R* implies greater influence on the results.

According to Table 4, the optimal parameter combination corresponds to the minimum *K* values (highlighted in bold): npop=30, rT=0.3, and ζ=0.05. Furthermore, the *R* values reveal that the population size (npop) has the most significant impact on the algorithm’s performance (*R* = 0.0033), followed by crossover intensity (*R* = 0.0031) and teacher ratio (*R* = 0.0028). These optimal parameters are adopted for all subsequent experiments.

### 5.2 Effectiveness validation

1) Effectiveness validation of the proposed strategies

To validate the necessity and contribution of the proposed knowledge-driven strategies, this paper designed two comparative variants, named KTLBO1 and KTLBO2. KTLBO1 is a version of KTLBO without the crossover-based teaching-learning operatorto simulate the lack of structural topology preservation, while KTLBO2 is a version of KTLBO without the neighborhood searchto simulate the absence of critical-path guidance. The Wilcoxon rank-sum test is employed to verify the significance of differences between optimal and general results. The results of the effectiveness experiment using evaluation metrics are presented in Table 5. The best values within the same category are displayed in bold, and the symbols “+”, “≈”, and “-” indicate that KTLBO is significantly better than, statistically equivalent to, and significantly worse than its rival methods.

**Table 5:** Statistical results of evaluation metrics by KTLBO with its variants.

Case	GD			Δ			IGD		
	KTLBO	KTLBO1	KTLBO2	KTLBO	KTLBO1	KTLBO2	KTLBO	KTLBO1	KTLBO2
#DP13	**0.0048**	0.4534(+)	0.205(+)	**0.8745**	0.9728(+)	0.8974(≈)	**0.0077**	0.493(+)	0.1499(+)
#DP14	**0.0065**	0.0525(+)	0.0068(≈)	**0.6661**	0.9137(+)	0.6866(≈)	**0.0181**	0.3376(+)	0.0185(≈)
#DP15	**0.0038**	0.1498(+)	0.0056(+)	**0.693**	0.9427(+)	0.7007(≈)	**0.0099**	0.2587(+)	0.0112(+)
#DP16	**0.0041**	0.4081(+)	0.0207(+)	**0.941**	0.9737(≈)	0.9616(≈)	**0.0641**	0.4729(+)	0.0799(+)
#DP17	0.0036	0.1511(+)	**0.0027**(≈)	0.8346	0.9362(+)	**0.8291**(≈)	0.0118	0.2819(+)	**0.0111**(≈)
#DP18	**0.0041**	0.0935(+)	0.0188(+)	**0.8644**	0.9551(≈)	0.87(≈)	**0.0102**	0.2836(+)	0.0128(+)
#MK06	**0.0006**	0.0009(+)	0.0007(≈)	**0.5443**	0.718(+)	0.6981(+)	**0.0079**	0.023(+)	0.008(≈)
#MK08	**0.0042**	0.056(+)	0.0128(+)	**0.8197**	0.9577(+)	0.8731(≈)	**0.0076**	0.0807(+)	0.0142(+)
#MK09	**0.0004**	0.0129(+)	0.0011(+)	**0.6031**	0.883(+)	0.632(≈)	**0.0008**	0.052(+)	0.0086(+)
#MK10	**0.0006**	0.0013(+)	0.0008(+)	**0.6787**	0.8533(+)	0.7483(+)	**0.0031**	0.029(+)	0.0062(+)
#MK12	**0.0035**	0.0102(+)	0.0053(+)	0.8082	0.8844(+)	**0.7861**(≈)	**0.0021**	0.0433(+)	0.0113(+)
#MK13	**0.0012**	0.0056(+)	0.0026(+)	**0.7473**	0.881(+)	0.8186(+)	**0.0044**	0.0494(+)	0.0057(+)
#MK14	**0.0019**	0.0129(+)	0.0025(+)	0.7405	0.9296(+)	**0.7223**(≈)	**0.0025**	0.0309(+)	0.0058(+)
#MK15	**0.0007**	0.0079(+)	0.0033(+)	**0.7333**	0.9004(+)	0.8219(+)	**0.003**	0.0293(+)	0.0048(+)

It can be observed that in terms of GD and IGD, KTLBO achieved the best values in all test cases except for #DP17, and is significantly superior to KTLBO1 and KTLBO2 in most cases. Regarding Δ, KTLBO obtains the best values in all test cases except for #DP17, #MK12, and #MK14, and is significantly better than KTLBO1 in most cases. The analysis of these results indicates that the discrete recombination mechanism is crucial; without the crossover-based operator (KTLBO1), the algorithm fails to preserve excellent gene structures of scheduling sequences, leading to a significant deterioration in convergence accuracy. Furthermore, KTLBO2 exhibits premature convergence, validating that the neighborhood search utilizing critical path knowledge is essential for breaking the deadlocks caused by tool capacity constraints. In summary, the proposed strategies are not merely incremental additions but are proven to be essential for addressing the specific complexity of BFJSP-TA.

2) Comparative analysis of algorithm performance

**Fig 6:**
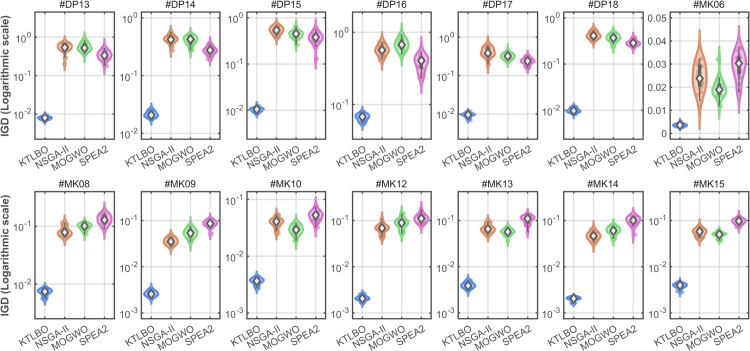
Violin plot of IGD metrics.

To verify the comprehensive performance of the proposed KTLBO algorithm, three well-known multi-objective meta-heuristics are employed as comparisons, which are NSGA-II [30], MOGWO [32], and SPEA2 [33]. The results of the comparative analysis of comprehensive algorithm performance using evaluation metrics are shown in Table 6. To further visualize the distribution and stability of the experimental results, the violin plots of the IGD metrics across all test cases are illustrated in Fig 6. To validate the statistical significance of the proposed method, the non-parametric Friedman test and Wilcoxon signed-rank test are conducted. The best values within the same category are displayed in bold, and the symbols “+”, “≈”, and “-” indicate that KTLBO is significantly better than, statistically equivalent to, and significantly worse than its comparison methods.

**Table 6:** Statistical results of evaluation metrics by KTLBO and its comparisons.

Case	GD				Δ				IGD			
	KTLBO	NSGA-II	MOGWO	SPEA2	KTLBO	NSGA-II	MOGWO	SPEA2	KTLBO	NSGA-II	MOGWO	SPEA2
#DP13	**0.0048**	0.4743(+)	0.4737(+)	0.4377(+)	**0.8745**	0.9571(+)	0.9552(+)	0.9798(+)	**0.0077**	0.5318(+)	0.5263(+)	0.3583(+)
#DP14	**0.0078**	0.1259(+)	0.1287(+)	0.2227(+)	**0.6661**	0.9585(+)	0.945(+)	0.9624(+)	**0.0223**	0.4497(+)	0.4283(+)	0.2644(+)
#DP15	**0.0038**	0.3422(+)	0.2424(+)	0.3726(+)	**0.693**	0.9653(+)	0.9685(+)	0.9673(+)	**0.0099**	0.533(+)	0.4234(+)	0.3605(+)
#DP16	**0.0041**	0.5054(+)	0.568(+)	0.4535(+)	**0.941**	0.9729(≈)	0.9735(≈)	0.9689(≈)	**0.0641**	0.5888(+)	0.6473(+)	0.3825(+)
#DP17	**0.0572**	0.3739(+)	0.298(+)	0.2902(+)	1.0158	0.958(≈)	**0.9568**(≈)	0.9593(≈)	**0.0095**	0.4155(+)	0.3278(+)	0.2485(+)
#DP18	**0.0253**	0.2398(+)	0.277(+)	0.3097(+)	0.9645	0.9615(≈)	**0.9214**(≈)	0.9631(≈)	**0.0099**	0.4462(+)	0.3649(+)	0.2901(+)
#MK06	**0.0006**	0.0007(+)	0.0008(+)	0.0164(+)	**0.6857**	0.8802(+)	0.8241(+)	0.8922(+)	**0.0036**	0.0213(+)	0.0191(+)	0.0291(+)
#MK08	**0.0042**	0.0561(+)	0.0774(+)	0.1686(+)	**0.8197**	0.9087(+)	0.9489(+)	0.9623(+)	**0.0076**	0.082(+)	0.0982(+)	0.127(+)
#MK09	**0.0006**	0.0092(+)	0.027(+)	0.0997(+)	**0.6262**	0.8979(+)	0.9207(+)	0.9465(+)	**0.0026**	0.0368(+)	0.0552(+)	0.0811(+)
#MK10	**0.0007**	0.0015(+)	0.0021(+)	0.0344(+)	**0.6787**	0.9263(+)	0.8615(+)	0.9082(+)	**0.0035**	0.0398(+)	0.0279(+)	0.0473(+)
#MK12	**0.0037**	0.0167(+)	0.0336(+)	0.1233(+)	**0.8082**	0.8966(+)	0.8798(≈)	0.8829(+)	**0.0022**	0.0696(+)	0.0832(+)	0.107(+)
#MK13	**0.003**	0.0061(+)	0.0059(+)	0.0627(+)	**0.7473**	0.9276(+)	0.9027(+)	0.9422(+)	**0.0041**	0.069(+)	0.0555(+)	0.1044(+)
#MK14	**0.0016**	0.0162(+)	0.0363(+)	0.1106(+)	**0.8323**	0.9148(+)	0.9392(+)	0.9376(+)	**0.002**	0.0465(+)	0.0614(+)	0.0954(+)
#MK15	**0.0007**	0.0109(+)	0.0048(+)	0.1004(+)	**0.7333**	0.9325(+)	0.908(+)	0.9493(+)	**0.004**	0.0561(+)	0.0456(+)	0.0892(+)

Table 7 presents the average rankings obtained from the Friedman test. It can be observed that KTLBO achieves the lowest ranking values (1.00, 1.29, and 1.00) across all three metrics (GD, *Δ*, and IGD), indicating the superior overall performance of the proposed algorithm. Furthermore, Table 8 summarizes the results of the Wilcoxon signed-rank test with a significance level of 0.05. The *p*-values for all pairwise comparisons are less than 0.05, which rejects the null hypothesis and confirms that the performance improvement of KTLBO is statistically significant.

**Table 7:** Average ranking values of algorithms by Friedman test.

Metric	KTLBO	NSGA-II	MOGWO	SPEA2
GD	1.00	2.79	2.64	3.57
*Δ*	1.29	2.86	2.64	3.21
IGD	1.00	3.07	2.50	3.43

**Table 8:** Statistics results by the Wilcoxon signed-rank test (α = 0.05).

Metric	Algorithm	+/(≈)/-	$R^{+}$	*R* ^*−*^ *-*	*p-value*	α = 0.05
GD	KTLBO vs. NSGA-II	14/0/0	105	0	0.0001	YES
	KTLBO vs. MOGWO	14/0/0	105	0	0.0001	YES
	KTLBO vs. SPEA2	14/0/0	105	0	0.0001	YES
*Δ*	KTLBO vs. NSGA-II	11/3/0	66	0	0.0012	YES
	KTLBO vs. MOGWO	10/4/0	55	0	0.0039	YES
	KTLBO vs. SPEA2	11/3/0	66	0	0.0012	YES
IGD	KTLBO vs. NSGA-II	14/0/0	105	0	0.0001	YES
	KTLBO vs. MOGWO	14/0/0	105	0	0.0001	YES
	KTLBO vs. SPEA2	14/0/0	105	0	0.0001	YES

In terms of GD and IGD, KTLBO achieved the best values in most instances and is significantly better than the comparative algorithms in almost all cases (14/0/0). As observed in Fig 6, the IGD distribution of KTLBO consistently lies at the lowest position with a more compact shape compared to other algorithms. This indicates that KTLBO not only achieves higher convergence accuracy but also demonstrates superior robustness and stability against random variations in independent runs. For Δ, KTLBO obtained the best values in all test cases except for #DP17 and #DP18, and is significantly superior to the comparative algorithms in most cases. Therefore, supported by the statistical tests, KTLBO demonstrates a clear advantage in comprehensive performance among state-of-art meta-heuristics.

Regarding the scalability of the algorithm, the test instances used in this study include varying scales (e.g., small-scale #MK06 and large-scale #DP18). As shown in Table 6 and the statistical tests, KTLBO maintains a consistent lead (Rank 1 and *p* < 0.05) across all instances regardless of the problem size. This suggests that the proposed knowledge-driven strategies can effectively handle the increased complexity of the solution space in larger problems, indicating good scalability of the proposed algorithm.

### 5.3 Comparison of KTLBO and Gurobi

To validate the correctness of the proposed MIP formulation for the BFJSP-TA and assess the capability of the KTLBO algorithm in obtaining high-quality solutions, the commercial exact solver Gurobi is employed as comparison. Due to the NP-hard nature of the problem, the comparison is conducted on a set of randomly generated small-scale instances (denoted as #Small1 to #Small5). In these instances, the processing times of operations are generated following a uniform distribution in the range [2,20].

Since Gurobi is designed primarily for single-objective optimization and cannot directly generate a Pareto front, the two objective functions of BFJSP-TA, namely Total Tool Wear Cost (*TWC*) and Weighted Sum of Tardiness (*WST*), are minimized separately to verify the algorithm’s accuracy in finding extreme values. KTLBO runs 20 independent times for each instance, and the best solution found is recorded. The time limit for Gurobi is set to 3600 seconds for each run to find the global optimum or a lower bound.

The comparison results are reported in Table 9. The performance gap is defined as in Eq. (19).

$$Gap(\%)=\frac{\text{KTLBO}-\text{Gurobi}}{\text{Gurobi}}\times 100\%$$ (19)

where a gap of 0 indicates that KTLBO successfully finds the same optimal solution as Gurobi. A positive gap implies that KTLBO finds a worse feasible solution than the continuous execution of Gurobi within the time limit. The superscript ^⋆^ marks values proven optimal by Gurobi.

**Table 9:** Comparison results of KTLBO and Gurobi on small-scale instances.

Instance	$\sqrt{\left|\text{M}|\times |\text{J}|\times |\text{O}|\times |\text{K}\right|}$ ^a^	*TWC*			*WST*		
		KTLBO	Gurobi	*Gap%*	KTLBO	Gurobi	*Gap%*
#Small1	3 × 3 × 9 × 4	88.26	88.26^⋆^	0	3.37	3.37^⋆^	0
#Small2	3 × 3 × 9 × 10	70.92	70.92^⋆^	0	0	0^⋆^	0
#Small3	4 × 4 × 13 × 6	112.81	111.46^⋆^	1.2	0.74	0.72^⋆^	2.8
#Small4	4 × 4 × 13 × 10	120.27	119.61	0.6	3.18	2.98	6.7
#Small5	4 × 5 × 15 × 10	134.02	130.70	2.5	1.17	1.04	12.5

^a^ |***M***| is the number of machines, |***J***| is the number of jobs, |***O***| is the total number of operations, and |K| is the number of tools.

As can be seen from Table 9, KTLBO demonstrates excellent convergence capabilities on small-scale instances. For the smallest instances (#Small1 and #Small2), KTLBO successfully finds the optimal solutions (Gap=0%) that are certified by Gurobi. For the slightly larger instances (#Small3 and #Small4), although the problem complexity increases, the gaps for the *TWC* objective remain strictly within 1.2%, and the gaps for the *WST* objective are kept within a reasonable range (2.8% to 6.7%). Even in instance #Small5, where the search space expands significantly, KTLBO maintains a competitive performance with a *TWC* gap of only 2.5%, while the *WST* gap inevitably increases slightly due to the conflict between objectives and the NP-hard nature of the problem. It is worth noting that KTLBO uses much less time in solving #Small3, #Small4 and #Small5. These results confirm that the proposed algorithm is capable of effectively approaching the global optimum, validating the reliability of its optimization mechanism.

### 5.4 Necessity of collaborative optimization

To validate the application effectiveness of the collaborative optimization of tool allocation and production scheduling proposed in this paper, a comparative experiment is conducted using the traditional sequential optimization method as a control. In the sequential optimization, tools are first randomly and evenly allocated to the machining equipment, followed by the optimization of operation assignment and machining sequence. To reduce the impacts of randomness, the sequential optimization experiments independently run five times (denoted as SO1-5). The experiments are carried out on test cases #DP13, #MK12, and #MK15.

**Fig 7:**
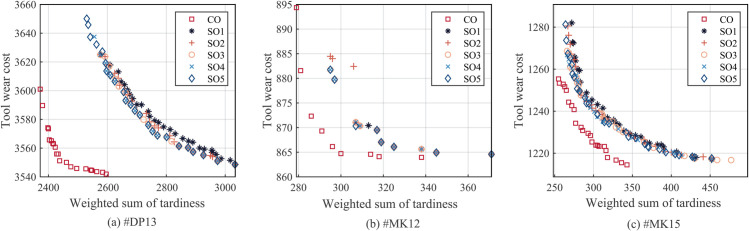
Pareto front obtained by collaborative optimization (CO) and sequential optimization (SO1-5).

As shown in Fig 7, the first front solutions obtained by the collaborative optimization method (CO) and the five sequential optimization methods (SO1-5) are depicted. It can be clearly seen that the front of CO completely dominates the front of SO.

The superiority of the collaborative strategy can be attributed to the inherent coupling between tool allocation and operation scheduling capabilities. In the sequential optimization framework, the decision space is artificially severed, as tool allocation is fixed prior to scheduling. This creates a resource rigidity constraint. For example, if the random allocation phase fails to assign a necessary tool to a high-performance machine, the subsequent scheduling phase is permanently blocked from utilizing that machine for the corresponding operation, even if it would have significantly reduced the tardiness. The scheduling algorithm is forced to search within a strictly limited subspace defined by the initial allocation.

Conversely, the collaborative optimization explores the joint search space of tool configuration and operation sequencing simultaneously. This allows the algorithm to adaptively resolve bottlenecks by reconfiguring tool locations to match scheduling needs, effectively assigning tools to machines that are identified as critical for minimizing delays or costs during the evolutionary process. The clear gap between the CO and SO fronts in Fig 7 visually quantifies the opportunity cost incurred by the sequential separation of these coupled decisions, demonstrating that high-quality solutions are structurally inaccessible to the sequential approach.

## 6 Conclusions and prospects

Tool allocation is a critical decision-making issue in the machine shop-based flexible manufacturing process. In this specific environment, the production efficiency is strictly constrained by the limited capacity of tool magazines and the inevitable degradation of tool life. This paper aims to reduce the tool wear cost and weighted sum of tardiness by a collaborative optimization method for tool allocation and production scheduling. Mathematically, the validity of the proposed methodology relies on specific boundary conditions: the accurate modeling of physical constraints (e.g., magazine capacity and tool compatibility) serves as the necessary condition for representing the real-world shop floor, while the algorithmic robustness and convergence stability serve as the sufficient conditions for ensuring that the simulated schedules are executable and reliable. A KTLBO algorithm is proposed to address the computational challenge inherent in satisfying these conditions. The results demonstrate that the proposed collaborative optimization method enhances the global optimal, thereby obtaining superior solutions. The KTLBO algorithm, introduced in this paper, has shown excellent solution performance and advantages when compared with state-of-art meta-heuristics.

The method presented in this paper incorporates essential practical constraints derived from manufacturing scenarios, providing a validated modeling and solving approach for intelligent decision-making in flexible workshops under realistic resource limitations. Future research can further extend to distributed manufacturing environments, taking into account the actual situations of actuator scheduling and completion time constraints.
